# ﻿*Argostemma sawmlianae* (Rubiaceae, Argostemmateae), a new species from Northeast India under the Indo-Burma Hotspot

**DOI:** 10.3897/phytokeys.266.162537

**Published:** 2025-11-25

**Authors:** Margaret Lalhlupuii, Lal Tlanhlui, Sandhyarani Devi Khomdram, Sanatombi Devi Yumkham

**Affiliations:** 1 Department of Botany, Mizoram University, Aizawl–796004, Mizoram, India Mizoram University Aizawl India; 2 Department of Life Sciences (Botany), Manipur University, Canchipur–795003, India Manipur University Canchipur India

**Keywords:** *
Argostemma
*, Indo-Burma hotspot, Mizoram, molecular, morphology, new species

## Abstract

*Argostemma sawmlianae* (Rubiaceae), a new species from Mizoram, Northeast India, within the Indo-Burma hotspot, is described and illustrated. The species is compared with its allied species *A. courtallense* and *A. sarmentosum*, from which it differs by a set of distinctive morphological characters that include a pair of larger sessile leaves (11.5–18.5 cm long), longer peduncle (4–6.5 cm long), more flowers per cyme (8–33) and campanulate corolla with falcate lobes, reflexed and prominently rolled backward. The molecular analyses based on *matK* (cpDNA) and ITS2 (nrDNA) sequences support the morphological evidences confirming *A. sawmlianae* as a distinct new species. The newly discovered species is provisionally categorized as Critically Endangered (CR) in accordance with the IUCN Red List Categories and Criteria (IUCN 2024) based on available data.

## ﻿Introduction

*Argostemma* Wall. (Wallich 1824: 324) is a genus that includes terrestrial and occasionally lithophytic or epiphytic herbaceous species. Most of the species are found widely distributed in tropical and sub-tropical Asia with the exceptions of *Argostemma africanum* K.Schum. (Schumann 1896: 423) and *A. pumilum* Benn. (Bennet 1838: 95) which are endemic to tropical Africa. Among the four subfamilies of Rubioideae under Rubiaceae, the tribe Argostemmateae, represents the largest genus with 220 described species (Verdcourt 1958; Bremer 1989; Sridith 2007; Bremer and Manen 2000). In India, the distribution of the genus is primarily confined to the Northeastern states and the Western Ghats which is evident from the reported cases of nine species so far, including the recent addition of *Argostemma quarantena* Balan & Robi (Balan et al. 2021: 48) and *A. kamjongense* Sadokpam, S.D.Khomdram & S.D.Yumkham (Sadokpam et al. 2023: 78). It may be noted that four species namely *A. verticillatum* Wall. (Wallich 1824: 325), *A. sarmentosum* Wall. (Wallich 1824: 324), *A. fragile* E. T. Geddes (Geddes 1927: 166), and *A. khasianum* C.B. Clarke (Clarke 1880: 43) have been reported so far from the state of Mizoram. (Choudhury 2002; Panday et al. 2020).

During June 2022 to October 2024, botanical field explorations in Mizoram led to the collection of an unidentified species of *Argostemma* from the forest of Lungleng village, situated about 20 km from Aizawl. Mizoram, one of the North-eastern states of India, is situated at the confluence of the Indo-Malayan and Indo-Chinese biogeographic regions, which are part of the Indo-Burma Biodiversity Hotspot, renowned for its rich floral diversity, including numerous endemic taxa. Based on morphological, palynological, molecular investigations, literature survey and herbarium specimen consultation, the Mizoram *Argostemma* plant was found to be an undescribed species. Molecular database of the newly described *Argostemma* species (*matK* and ITS2) was generated and phylogenetic analysis confirmed its novelty as a new species, and we hereby describe it as *Argostemma sawmlianae*.

## ﻿Materials and methods

### ﻿Morphological analyses

Specimens of an unidentified *Argostemma* were collected during extensive field surveys from Mizoram during 2022–2024. Measurements of all morphological characteristics of the new species were taken from fresh samples collected from the type locality. Relevant literature (such as Hooker 1880; Ridley 1923, 1927; Bremer 1989; Sridith 1999, 2007; Sridith and Puff 2000; Choudhury 2002; Chen et al. 2011; Panday et al. 2020) and *Argostemma* materials from several herbaria (ARUN, ASSAM, CAL) were consulted. Additionally, numerous digital specimens, including type and general herbarium specimens, were examined from online resources such as the Natural History Museum, London (BM; https://www.nhm.ac.uk) and the Royal Botanic Gardens, Kew (K; https://data.kew.org) to confirm the novelty of the species. High resolution specimen images were also consulted from major global databases, including the Global Biodiversity Information Facility (https://www.gbif.org), JSTOR Global Plants (http://plants.jstor.org), Missouri Botanical Garden-TROPICOS (https://tropicos.org), Plants of the World Online (https://powo.science.kew.org).

Terminology used in describing the new species follows Beentje (2016). The conservation status was assessed as per IUCN Red List Categories and Criteria (IUCN 2024). Photographs were taken by using DSC–W610 digital camera (Sony, Tokyo, Japan) and BT-E Benchtop biological digital microscope (Cilika, Thane, India). The palynological studies were done using fresh pollens and the size was expressed as Polar axis (P) × Equatorial axis (E) in micrometre (µm) (Schlag-Edler and Kiehn 2001). Terminologies given by Punt et al. (2007) and Halbritter et al. (2018) are used for describing the pollen characters. Seeds and pollen grains prepared for micromorphological analysis were dehydrated, subjected to critical point drying, and mounted on stubs coated with gold using Fine Coat Ion Sputter JFC-1100. The samples were subsequently examined and microphotographs were obtained using field emission scanning electron microscope (JEOL JSM-6360, Freising, Germany).

### ﻿Molecular analyses

Genomic DNA from the fresh sample of the newly described *Argostemma* was extracted using CTAB method (Doyle and Doyle 1987). PCR amplification was done using *matK* (cpDNA) primer (F- CGTACAGTACTTTTGTGTTTACGAG; R-ACCCAGTCCATCTGGAAATCTTGGTTC) and ITS2 (nrDNA) primer (F- ATGCGATACTTGGTGTGAAT; R- GACGCTTCTCCAGACTACAAT). The PCR product was sequenced using Sanger sequencing method. The determined *matK* and ITS2 sequences were deposited in the GenBank (www.ncbi.nlm.nih.gov/Genbank) of National Center for Biotechnology Information (NCBI) with accession No. OR555879 and PP067885 respectively. Sequences obtained for the submitted taxa and closely related species were aligned using the ClustalW algorithm implemented in MEGA X software (Cheng et al. 2022). A phylogenetic tree was constructed in MEGA X using the Maximum Likelihood method with the Kimura-2-Parameter model and a discrete Gamma distribution, supported by 1000 bootstrap replicates. The best-fit nucleotide substitution model was selected based on the lowest BIC score. Twenty-nine *matK* sequences of thirteen genera and twenty-five ITS2 sequences of twelve genera were obtained from GenBank and used in phylogenetic analyses, with *Cinchona officinalis* L. as outgroup. The resulting tree was analysed to determine the placement of the sequences about the known taxa. The NCBI accession numbers of the Rubiaceae members including the outgroup used in this study are detailed in Suppl. material 1: table S1.

## ﻿Taxonomy

### ﻿ Argostemma sawmlianae

Taxon classificationPlantaeGentianalesRubiaceae

Lalhlupuii, Tlanhlui, S.D.Khomdram & S.D.Yumkham sp. nov.

DA3B4D69-11EB-55A9-BB70-64038CB0C553

urn:lsid:ipni.org:names:77363110-1

Figs 1, 2, 3

#### Type.

India. • Mizoram, Aizawl district, Lungleng village, 23.66581, 92.66353, 1003 m alt., 05 July 2022, *Lalhlupuii 128870* (holotype: ASSAM!; isotype: MZUH!, MUMP!).

#### Diagnosis.

It is similar to *Argostemma courtallense* Arn. and *A. sarmentosum* Wall., but differs from the two in having one pair of larger sessile leaves (11.5–18.5 cm long), longer peduncle (4–6.5 cm long), more flowers per cyme (8–33), and campanulate corolla with falcate petals which are reflexed and strongly rolled backwards (Fig. 3, Table 1).

**Table 1.** Comparison of *Argostemma sawmlianae*, *A. courtallense* and *A. sarmentosum*.

Characters	*A. sawmlianae*	*A. courtallense*	*A. sarmentosum*
Rhizomes	Non-tuberous	Tuberous	Tuberous
Stem size	1.5–7 cm	8–20 cm	10–20 cm
Leaves	1-pair, anisophyllous, broadly ovate to elliptic, 11.5–18.5 cm long	2-pairs, isophyllous, elliptic ovate, 7 cm long	2-pairs, anisophyllous, elliptic ovate, 2–10 cm long
Stipule	Ovate, 5–7 × 3–4 mm	Ovate, 7–8 × 1–2 mm	Broadly ovate or elliptic, 1.5–2 × 2 mm
Petiole	Sessile	0–2 cm	0.2–0.3 cm
Number of flowers per cyme	8–33 flowered	3 to many flowered	6–10 flowered
Bract	4–6, basally connate	Free	2–3, free
Peduncle	4–6.5 cm	3–6 cm	3–6 cm
Calyx	Lobes 4, 6–10 × 4–5 mm, sequential colour changes from white to green	Lobes 4, 3 × 2 mm, sequential colour changes from white to green	Lobes 5, 9–10 × 4–5 mm long, persistent green
Corolla	Campanulate, lobes falcate, 7–10 × 3–6 mm, reflexed and strongly rolled backwards	Star shaped, lobes ovate, 7–8 × 2–2.5 mm, not reflexed	Star shaped, lobes narrowly triangular, 7–8 × 2.5–3 mm, rarely reflexed
Stamen	Filament 2–3 mm, anther 6 mm	Flament 3 mm, anther 3 mm	Filament 2.5–2.8 mm, anther 6 mm

#### Description.

Epilithic, perennial herb, 7–17 cm tall, attached to substratum with dense, much-branched, non-tuberous matted roots. Stem semi-erect, terete, unbranched, 1.5–7 cm long, greenish white, pubescent. Leaves opposite, strongly anisophyllous, paired, sessile, reticulate eucamptodromous, secondary veins 6, ovate, 11.5–18.5 × 10–13.3 cm, base cordate, margin entire, apex acute, pubescent on both sides; small pair of rudimentary leaves, sessile, broadly ovate to elliptic, 3–7 × 3–4 mm, green, pubescent. Stipule sessile, ovate, 5–7 × 3–4 mm, base cordate, apex acute, green, margin pubescent. Inflorescence umbelliform, flowers basipetal, single, rarely 2–4, 8–33 flowered. Bracts foliaceous, 4–6, fused, unequal size, whorled, elliptic to ovate, 8–11 × 4–6 mm, green, margin pubescent, whorled, isomorphic with number of inflorescences (1–4); peduncle 4–6.5 cm long, greenish white, glabrous. Flowers pedicellate, 4-merous, actinomorphic, 1.8–2.2 cm, pedicel 7–10 mm long, white, pubescent. Calyx 4-lobed, lobes basally connate, ovate, 6–10 × 4–5 mm, petaloid, white, calyx teeth tip green, become completely green after anthesis, glabrous. Corolla 4, white, falcate, 6–8 × 2–3 mm; ¾ lobes reflexed, tube green blotched, 1–2 × 1–3 mm, tuff haired at tip of corolla lobe. Stamens 4, equal, anthers free, loosely arranged, basifixed, 6 mm long, yellow, exserted from the corolla tube, glabrous; filaments 2–3 mm, free, S-like curvature, white.

**Figure 1.**
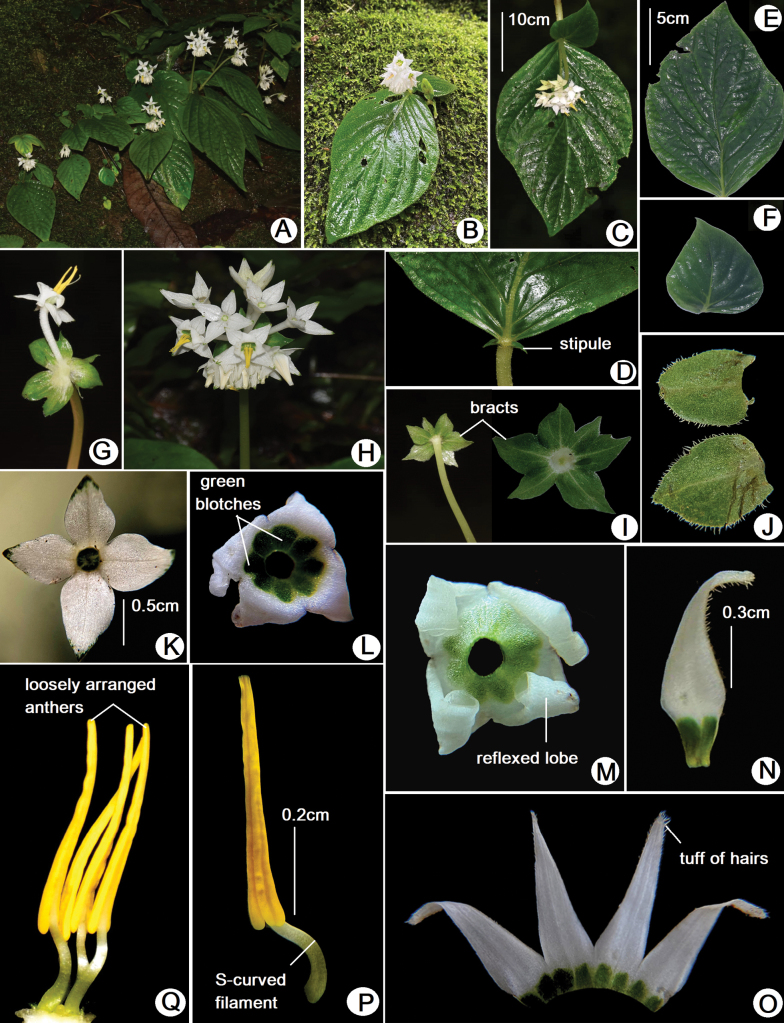
*Argostemma sawmlianae* Lalhlupuii, Tlanhlui, S.D.Khomdram & S.D.Yumkham, sp. nov. **A, B.** Habit; **C.** Anisophyllous leaves with inflorescence (inverted view); **D.** Leaf with stipule; **E, F.** Anisophyllous leaves; **G.** Flower with bract; **H.** Inflorescence; **I.** Foliaceous bracts; **J.** Rudimentary leaves; **K.** Petaloid calyx with green tips; **L.** Corolla with green blotched tubes; **M.** Corolla with reflexed and backwardly rolled lobes; **N.** Single falcate shaped corolla lobe; **O.** Corolla lobes; **P.** Single stamen with curved filament; **Q.** Androecium with loosely arranged stamens. (photos by Margaret Lalhlupuii & Lal Tlanhlui).

**Figure 2.**
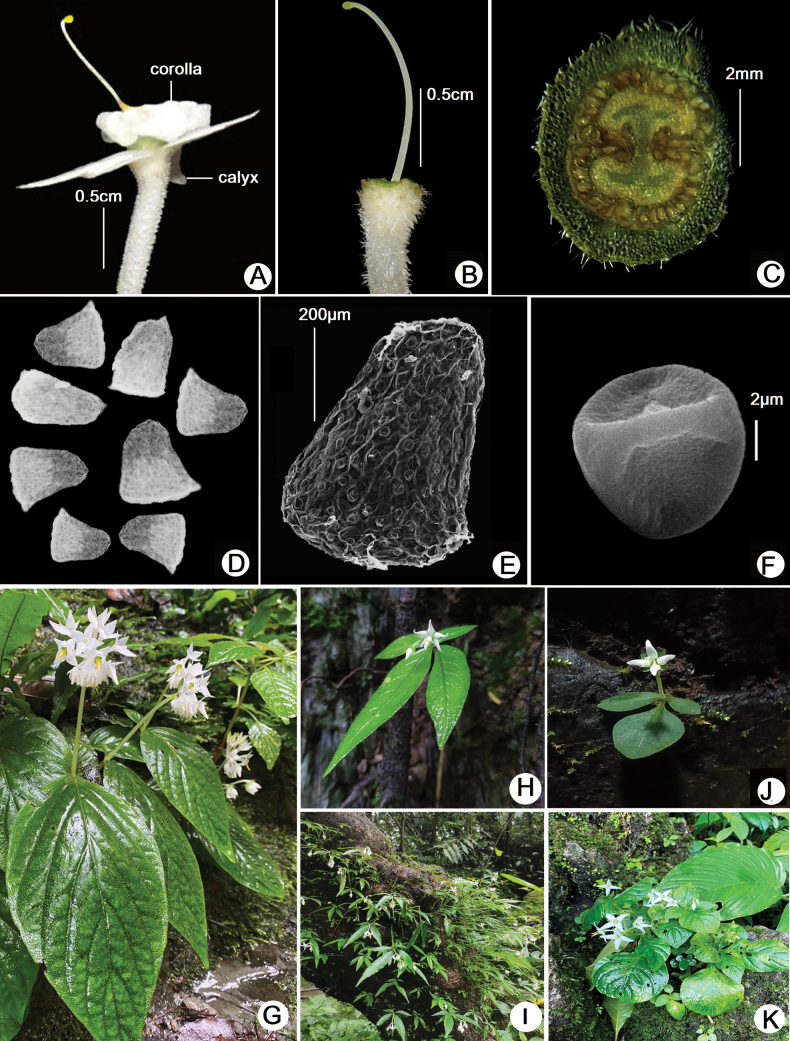
*Argostemma sawmlianae* Lalhlupuii, Tlanhlui, S.D.Khomdram & S.D.Yumkham, sp. nov. **A.** Single flower; **B.** Pistil; **C.** Cross section of ovary; **D.** Seeds (LM image); **E.** Seed (SEM image); **F.** Pollen (SEM image); **G.***A. sawmlianae* in type locality; **H, I.***A. verticillatum* from Manipur; **J.***A. kamjongense* from Manipur; **K.***A. sarmentosum* from Meghalaya. (photos by Margaret Lalhlupuii, Lal Tlanhlui & Yumkham).

Style 1–1.2 cm long, white, glabrous; stigma club shaped, yellow; ovary inferior, bilocular, axile placentation, hairy; ovule numerous, yellow. Seeds numerous, minute, triangular, 600 × 300 μm, black, testa reticulate, central mamilla in each cavity (Fig. 2D, E).

**Figure 3.**
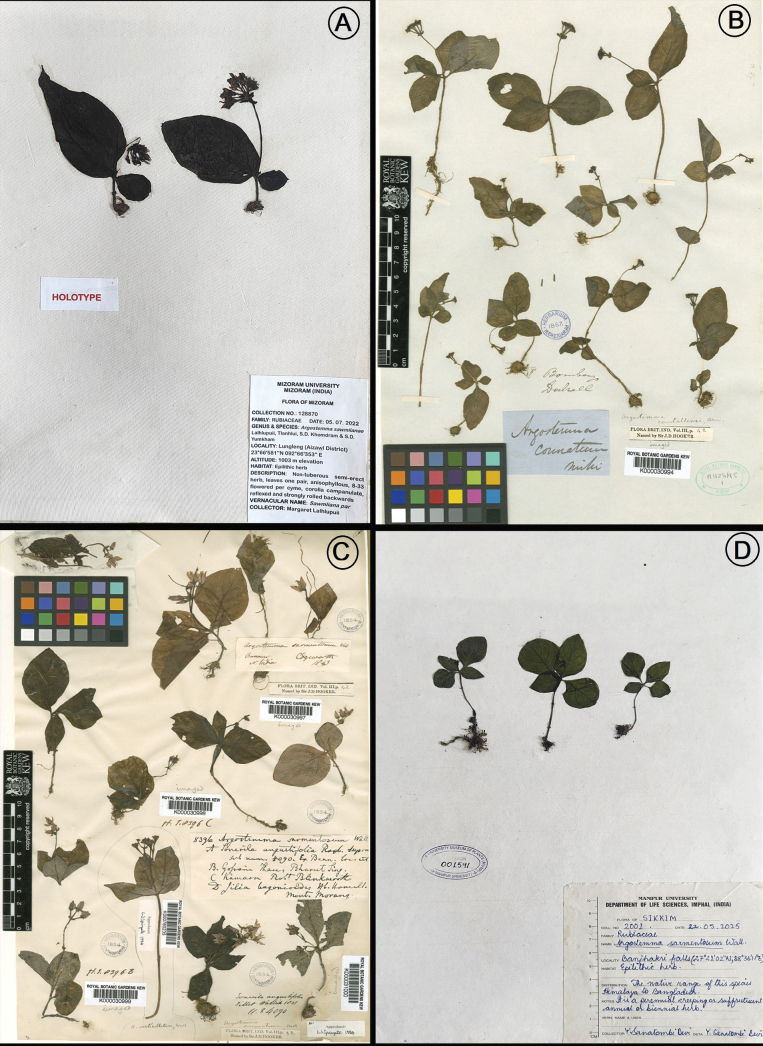
**A.** Holotype of *Argostemma sawmlianae* Lalhlupuii, Tlanhlui, S.D.Khomdram & S.D.Yumkham, sp. nov. (Lalhlupuii 128870, ASSAM!); **B.** Herbarium Specimen of *A. courtallense* [K000030994] (reproduced with permission from http://specimens.kew.org/herbarium/K000030994); **C.** Herbarium specimen of *A. sarmentosum* [K000030998] (reproduced with permission from http://specimens.kew.org/herbarium/K000030998); **D.** Herbarium specimen of *A. sarmentosum* [MUMP001591]

#### Pollen grains.

Monad, heteropolar, very small 8–9 × 8–9 µm, triangular amb, isodiametric (P/E ratio 1), triradiate colpi (dry) (Fig. 2F).

#### Phenology.

Flowering from June–July and fruiting from July–August.

#### Distribution and ecology.

The plant was found growing on a damp rock-wall along a stream with mosses, pteridophytes (*Selaginella* sp., *Asplenium* sp.) and *Begonia* spp. (Begoniaceae). The plant was collected from *Lungleng* village in Aizawl District of Mizoram, India which is located at 23°39'57"N, 92°39'41"E, south of Aizawl city. Positioned at an elevation of 1011 m above sea level, Lungleng receives an average annual rainfall of about 2500 mm, with temperature ranging from 26 °C–31 °C. The settlement lies atop a hill and is encircled by a narrow belt of dense community reserve forest comprising subtropical broad-leaved evergreen mixed forest and with patches of secondary forest surrounding the reserved area.

#### Conservation status.

The newly described species was found growing as a lithophyte at its type locality only, represented by a single population of 39 mature individuals which are less than 50. The habitat which is situated along a roadside faces significant threats from frequent landslides and ongoing developmental activities such as road construction and expansion. The estimated area of occupancy (AOO) is less than 10 km^2^. Based on current field observations, the species is provisionally assessed as Critically Endangered [CR B2 ab (ii, iii, v) c (ii, iii, iv) D] according to the IUCN Red List Categories and Criteria (IUCN 2024). Further research is necessary for thorough and long-term population assessment to yield a more precise conservation evaluation.

#### Etymology.

The specific epithet *sawmlianae* is to honour Mal Sawmliana, a forest conservator and naturalist, as a recognition of his efforts and contribution in exploring the flora and fauna for conservation measures in Mizoram.

#### Suggested common name.

*Sawmliana par* (Mizo)

##### ﻿Molecular studies of *Argostemma sawmlianae*

**Figure 4.**
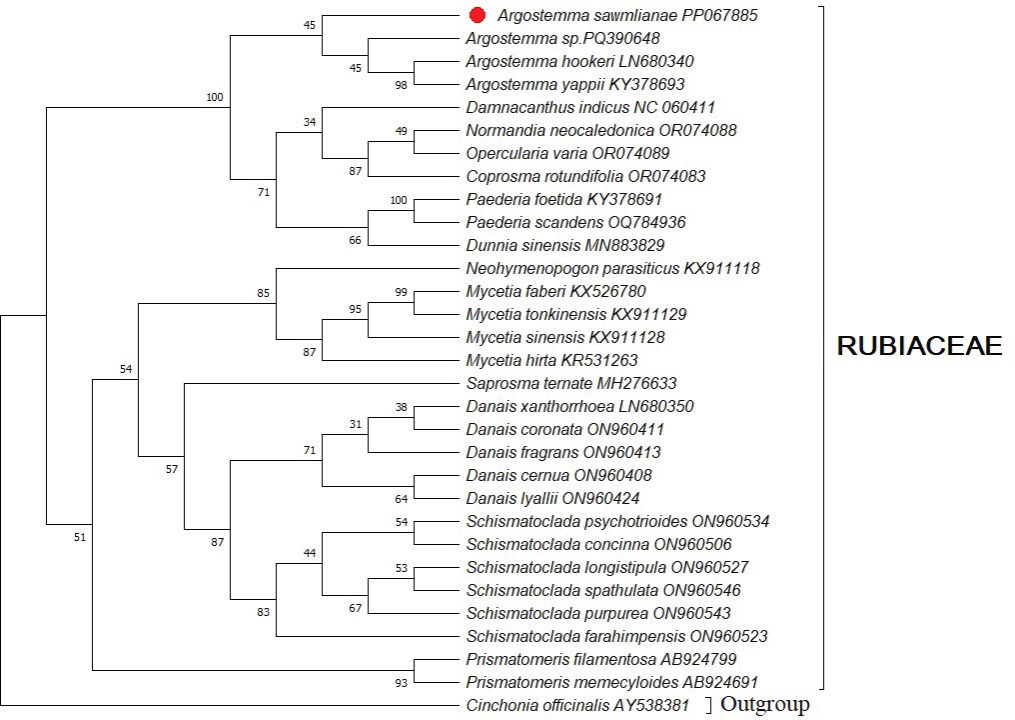
Phylogenetic tree of *Argostemma sawmlianae* reconstructed based on Maximum Likelihood (ML) as a cladistic method comprising 31 *matK* nucleotide sequences using MEGA X. Red solid circle indicates the placement of *A. sawmlianae* among *A. hookeri*, *A. yappi* and *Argostemma* sp. in the phylogenetic tree.

**Figure 5.**
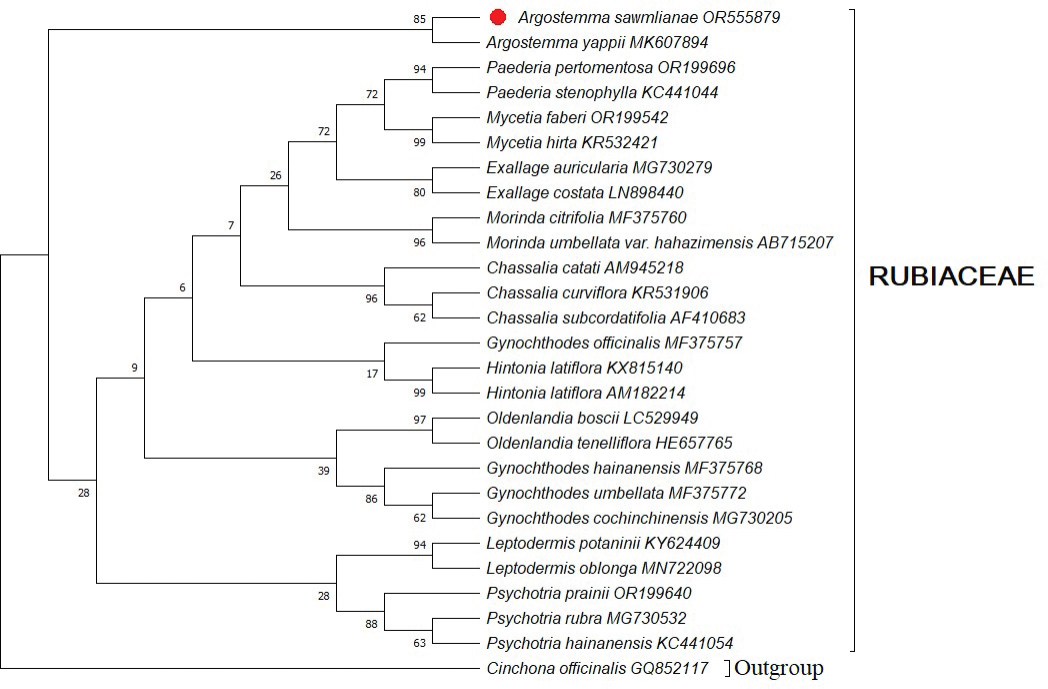
Phylogenetic tree of *Argostemma sawmlianae* reconstructed based on Maximum Likelihood (ML) as a cladistic method comprising of 27 ITS2 nucleotide sequences using MEGA X. Red solid circle indicates the placement of *A. sawmlianae* with *A. yappi* in the phylogenetic tree.

After analysis of the *matK* and ITS2 sequences from *Argostemma sawmlianae*, it was observed that the consensus length of the *matK* sequence is 766 bp with 29.51% G:C content and that of ITS2 is 438 bp with 52.69% G:C content. A Maximum Likelihood (ML) tree was constructed using *matK* and ITS2 sequences in MEGA X (Molecular Evolutionary Genetics Analysis, X) to analyze the placement of *A. sawmlianae* among different members of Rubiaceae with *Cinchona officinalis* as an outgroup species (Figs 4, 5).

Phylogenetic analyses based on *matK* and ITS2 regions of the newly described species and available database from NCBI (Suppl. material 1: table S1) confirmed the placement of the newly described species within *Argostemma* (Rubiaceae). These findings align with Bremer (2009), who established the monophyly of the Rubiaceae family. The *matK* phylogenetic tree shows that *A. sawmlianae* clusters closely with *A. hookeri* King and *A. yappii* King, supported by bootstrap values ranging from 45 to 98. Similarly, in the ITS2 phylogenetic tree, *A. sawmlianae* forms a well-supported clade with *A. yappii* (bootstrap 85), together constituting a distinct *Argostemma* group. The genomic data for allied taxa *Argostemma courtallense* Arn. and *A. sarmentosum* Wall. were not available in the NCBI database, and therefore, these species could not be included in the phylogenetic analysis.

## ﻿Discussion

The comparative analyses based on morphological characters confirm that *Argostemma sawmlianae* sp. nov. is morphologically distinct from closely related species such as *A. courtallense* and *A. sarmentosum* (Table 1). Key differentiating traits include its short semi-erect stem, large anisophyllous pair of leaves, more number of flower count per cyme, uniquely connate and foliaceous bracts, glabrous peduncle and its distinctive campanulate corolla with reflexed, falcate lobes. These consistent and non-overlapping characteristics support its recognition as a novel species within the genus *Argostemma*.

## Supplementary Material

XML Treatment for Argostemma sawmlianae

## ﻿Additional information

### Use of AI

No use of AI was reported.

### Author ORCIDs

Margaret Lalhlupuii https://orcid.org/0009-0009-4777-164X

Lal Tlanhlui https://orcid.org/0009-0008-9757-3978

Sandhyarani Devi Khomdram https://orcid.org/0000-0002-4084-9637

Sanatombi Devi Yumkham https://orcid.org/0000-0001-6191-0970

### Data availability

All of the data that support the findings of this study are available in the main text and GenBank data of NCBI accession numbers shown in Suppl. material 1: table S1.

## Supplementary materials

Supplementary material 1 Accession numbers of *matK* and ITS2 gene sequences information of Rubiaceae species Data type docx Explanation note Downloaded from the GenBank (www.ncbi.nlm.nih.gov/Genbank). https://binary.pensoft.net/file/1477627This dataset is made available under the Open Database License (http://opendatacommons.org/licenses/odbl/1.0/). The Open Database License (ODbL) is a license agreement intended to allow users to freely share, modify, and use this Dataset while maintaining this same freedom for others, provided that the original source and author(s) are credited.Margaret Lalhlupuii, Lal Tlanhlui, Sandhyarani Devi Khomdram, Sanatombi Devi Yumkham
