# Supplementary material for: ﻿Argostemma sawmlianae (Rubiaceae, Argostemmateae), a new species from Northeast India under the Indo-Burma Hotspot
Source: PhytoKeys. 2025 Nov 25;266:317–28. doi: 10.3897/phytokeys.266.162537 (PMC12673332; doi:10.3897/phytokeys.266.162537)
Supplement: Supplementary material 1 — Accession numbers of matK and ITS2 gene sequences information of Rubiaceae species [file phytokeys-266-317_article-162537__-s001.docx]

Supplementary Material Table S1. Accession numbers of *matK* and ITS2 gene sequences information of Rubiaceae species downloaded from the GenBank ([www.ncbi.nlm.nih.gov/Genbank](http://www.ncbi.nlm.nih.gov/Genbank))

| No. | Species | *matK* | ITS2 |
| --- | --- | --- | --- |
| 1 | *Argostemma sawmlianae* sp. nov. | PP067885 | OR555879 |
| 2 | *Argostemma sp.* | PQ390648 | - |
| 3 | *Argostemma hookeri* King | LN680340 | - |
| 4 | *Argostemma yappi* King | KY378693 | MK607894 |
| 5 | *Chassalia catati* Drake ex Bremek. | - | AM945218 |
| 6 | *Chassalia curviflora* (Wall.) Thwaites | - | KR531906 |
| 7 | *Chassalia subcordatifolia* (De Wild.) Piesschaert | - | AF410683 |
| 8 | *Coprosma rotundifolia* A.Cunn. | OR074083 | - |
| 9 | *Damnacanthus indicus* C.F.Gaertn. | NC060411 | - |
| 10 | *Danais cernua* Baker | ON960408 | - |
| 11 | *Danais coronata* (Pers.) Steud. | ON960411 | - |
| 12 | *Danais fragrans* (Lam.) Pers. | ON960413 | - |
| 13 | *Danais lyallii* Baker | ON960424 | - |
| 14 | *Danais xanthorrhoea* (K.Schum.) Bremek. | LN680350 | - |
| 15 | *Dunnia sinensis* Tutcher | MN883829 | - |
| 16 | *Exallage Auricularia* (L.) Bremek. | - | MG730279 |
| 17 | *Exallage costata* (Roxb.) Bremek. | - | LN898440 |
| 18 | *Gynochthodes cochinchinensis* (DC.) Razafim. & B.Bremer | - | MG730205 |
| 19 | *Gynochthodes hainanensis* (Merr. & F.C.How) Razafim. & B.Bremer | - | MF375768 |
| 20 | *Gynochthodes officinalis* (F.C.How) Razafim. & B.Bremer | - | MF375757 |
| 21 | *Gynochthodes umbellata* (L.) Razafim. & B.Bremer | - | MF375772 |
| 22 | *Hintonia latiflora* (Sessé & Moc. ex DC.) Bullock | - | KX815140 |
| 23 | *Hintonia latiflora* (Sessé & Moc. ex DC.) Bullock | - | AM182214 |
| 24 | *Leptodermis oblonga* Bunge | - | MN722098 |
| 25 | *Leptodermis potaninii* Batalin | - | KY624409 |
| 26 | *Morinda citrifolia* L. | - | MF375760 |
| 27 | *Morinda umbellate var. hahazimensis* L. | - | AB715207 |
| 28 | *Mycetia faberi* (Hemsl.) Razafim. & B.Bremer | KX526780 | OR199542 |
| 29 | *Mycetia hirta* Hutch. | KR531263 | KR532421 |
| 30 | *Mycetia sinensis* (Hemsl.) Craib | KX911128 | - |
| 31 | *Mycetia tonkinensis* (Pit.) Razafim. & B.Bremer | KX911129 | - |
| 32 | *Neohymenopogon parasiticus* (Wall.) Bennet | KX911118 | - |
| 33 | *Normandia neocaledonica* Hook.f. | OR074088 | - |
| 34 | *Oldenlandia boscii* (DC.) Chapm. | - | LC529949 |
| 35 | *Oldenlandia tenelliflora* (Blume) Kuntze | - | HE657765 |
| 36 | *Opercularia varia* Hook.f. | OR074089 | - |
| 37 | *Paederia foetida* L. | KY378691 | - |
| 38 | *Paederia pertomentosa* Blume | - | OR199696 |
| 39 | *Paederia scandens* (Lour.) Merr. | OQ784936 | - |
| 40 | *Paederia stenophylla* Merr. | - | KC441044 |
| 41 | *Prismatomeris filamentosa* Craib | AB924799 | - |
| 42 | *Prismatomeris memecyloides* Craib | AB924691 | - |
| 43 | *Psychotria hainanensis* H.L.Li | - | KC441054 |
| 44 | *Psychotria prainii* H.Lév. | - | OR199640 |
| 45 | *Psychotria rubra* (Lour.) Poir. | - | MG730532 |
| 46 | *Saprosma ternata* (Wall.) Benth. & Hook.f. ex Kurz | MH266633 | - |
| 47 | *Schismatoclada concinna* Baker | ON960506 | - |
| 48 | *Schismatoclada psychotriodes* Baker | ON960534 | - |
| 49 | *Schismatoclada longistipula* Cavaco | ON960527 | - |
| 50 | *Schismatoclada spathulate* D.Strid & Razafim. | ON960546 | - |
| 51 | *Schismatoclada purpurea* Homolle | ON960543 | - |
| 52 | *Schismatoclada farahimpensis Homolle* | ON960523 | - |
| Outgroup | | | |
| 53 | *Cinchona officinalis* L. | AY538381 | GQ852117 |
